# Role of Collagen in Airway Mechanics

**DOI:** 10.3390/bioengineering8010013

**Published:** 2021-01-16

**Authors:** Lumei Liu, Brooke Stephens, Maxwell Bergman, Anne May, Tendy Chiang

**Affiliations:** 1 Center of Regenerative Medicine, Abigail Wexner Research Institute, Nationwide Children’s Hospital, Columbus, OH 43215, USA; Lumei.Liu@nationwidechildrens.org; 2 College of Medicine, The Ohio State University, Columbus, OH 43210, USA; Brooke.Stephens@osumc.edu; 3 Department of Otolaryngology-Head & Neck Surgery, The Ohio State University Wexner Medical Center, Columbus, OH 43210, USA; Maxwell.Bergman@osumc.edu; 4 Section of Pulmonary Medicine, Nationwide Children’s Hospital, Columbus, OH 43205, USA; Anne.May@nationwidechildrens.org; 5 Department of Pediatrics, The Ohio State University Wexner Medical Center, Columbus, OH 43205, USA; 6 Department of Pediatric Otolaryngology, Nationwide Children’s Hospital, Columbus, OH 43205, USA

**Keywords:** collagen, airway mechanics, stiffness, airway disease

## Abstract

Collagen is the most abundant airway extracellular matrix component and is the primary determinant of mechanical airway properties. Abnormal airway collagen deposition is associated with the pathogenesis and progression of airway disease. Thus, understanding how collagen affects healthy airway tissue mechanics is essential. The impact of abnormal collagen deposition and tissue stiffness has been an area of interest in pulmonary diseases such as cystic fibrosis, asthma, and chronic obstructive pulmonary disease. In this review, we discuss (1) the role of collagen in airway mechanics, (2) macro- and micro-scale approaches to quantify airway mechanics, and (3) pathologic changes associated with collagen deposition in airway diseases. These studies provide important insights into the role of collagen in airway mechanics. We summarize their achievements and seek to provide biomechanical clues for targeted therapies and regenerative medicine to treat airway pathology and address airway defects.

## 1. Introduction

The airway consists of both a conducting region (larynx, trachea, bronchi, bronchioles) where air is humidified, warmed, and cleaned and a respiratory zone where gas exchange occurs. The airway is directly and continuously exposed to both macromechanical and micromechanical forces. Macromechanics is the study of organ-level mechanical and material properties. Intrathoracic respiratory forces, perfusion, and cough represent some of the dynamic macromechanical forces exerted on the respiratory system. As the airway is composed of heterogeneous components (chondrocytes, epithelium, endothelium, muscle, extracellular matrix (ECM)), these constituents can be individually quantified using micromechanics. Micromechanical properties drive the mechanotransduction in the airway, driving cell–cell and cell–matrix interactions [1].

The collagen family is the most abundant component of the airway ECM [2,3,4,5], providing structural support and facilitating cell adhesion and tissue development [6]. Diverse collagen subtypes are represented throughout the airway: Type IV collagen is the chief component of the basement membrane [7], type II collagen predominates in airway cartilage, and type I and III collagen are found in the alveolar wall and alveolar septa [8]. Due to their abundance in the alveoli, type I and III collagen are the primary contributors to lung mechanics [2,7,9]. Collagen homeostasis is dynamic and can be influenced by injury, repair, and pathologic change [10,11,12]. As a result, byproducts from collagen synthesis and degradation can serve as biomarkers for disease progression.

In this review, we appraise the role of collagen on normal airway mechanics. We then present studies of airway biomechanics, including the quantification of (1) airway micromechanics (organ-level structural properties) and (2) micromechanical properties (cell- and matrix-level properties). In the last section, we review the role of pathologic collagen deposition and alteration in airway diseases and its resultant effect on airway mechanics. As collagen of airway ECM is becoming a primary biological source for airway tissue engineering, understanding its impact on airway mechanics will facilitate the development of biomaterials that can recapitulate the native airway.

## 2. Collagen Determines Airway Mechanics

The primary role of collagen is to provide tensile strength to the ECM [13,14], with collagen subtypes assuming different roles in airway tissue (Table 1). With 28 different subtypes of collagen, subtypes I, II, and III predominate, representing 80~90% of total collagen [15,16]. In the airway ECM, type I collagen provides mechanical stability and structure. Type II collagen is the major component of airway cartilage (95% of total collagen), facilitating chondrocyte synthesis of ECM [17,18,19]. Type I and III collagens provide structural framework in the bronchi, interstitium, and alveolar wall [20,21,22]. Type III collagen in the airway is flexible, existing as narrow fibrils, and is more susceptible to breakdown than other fibrillar collagens [10,12,13,23]. Together, the collagen type I / type III ratio determines the resistance of collagen fibers to breakdown under mechanical forces during stretching [2]. Type IV collagen fiber is fundamental for maintaining the strength and function of the basement membranes [24,25].

As the primary structural component of airway ECM, collagen provides biomechanical cues for cell adhesion and tissue growth [6]. Studies of ECM mechanics in pulmonary diseases suggest that collagen is the most important load-bearing component of the lung parenchyma and has an essential role in maintaining tissue homeostasis and mediating cellular responses to injury [2]. Mechanical cues within collagen matrices serve to organize cell arrangement in the ECM: these cues facilitate cell alignment and cell–matrix bundling of collagen; conversely, pathologic changes in collagen fibril formation can prevent cell alignment and cell polarity [26].

Collagens also play a vital role during airway regeneration and repair. Regenerative medicine has adopted the use of acellular airway constructs through decellularization in an effort to provide a biomimetic scaffold for tissue engineering. However, decellularization of a multi-lineage tissue (bearing epithelial, vascular, muscle, and cartilaginous structures) such as the trachea has resulted in ECM injury, loss of graft mechanical properties, and collapse in both pre-clinical and clinical applications [27,28]. New approaches to tracheal tissue engineering have focused on the preservation of the native ECM, most importantly its collagen content [29,30].

## 3. Approaches to Quantify Airway Mechanics

### 3.1. Macromechanical Properties of the Airway

Airway mechanics has been quantified at macro and micro levels (Table 2) to explore the effect of the structural organization and micro-environment on airway function. On macro scale, the airway must retain patency and rigidity to perform normal respiratory function, and maintain elasticity to account for movement. These properties are most commonly assessed with ex vivo tensile and compression testing. This has been performed in the several animal models, defining normal tensile strength and elasticity [31,32,33,34]. Furthermore, uniaxial tensile testing has also been used to establish criteria for decellularization [35,36]. Preservation of graft macromechanical properties can serve as a surrogate for ECM preservation (e.g., collagen) following modulation of native tissues.

In contrast, several modalities are used at the bedside to assess macromechanical properties of the airway in vivo. Functional assessment of the airway can be achieved quantitatively with the use of pulmonary function testing (PFT), for example with plethysmography to calculate lung volumes and spirometry to evaluate for the presence of intrathoracic or extrathoracic obstruction. Beyond pulmonary function tests, radiographic tests can be used to characterize changes in tissue thickness [37]. Using a Jacobian determinant measured from computed tomography (CT), biomechanical metrics representing local lung expansion and contraction can predict respiratory morbidity and mortality in chronic obstructive pulmonary disease (COPD) [38]. Dynamic imaging of the airway permits the quantification of airway diameter during spontaneous respiration; techniques such as dynamic volumetric computed tomographic angiography (DV-CTA) of the chest and airway fluoroscopy are used to assess for airway collapse seen in tracheobroncheomalacia [39]. Under anesthesia, bronchoscopy allows for the direct visualization of the tracheobronchial airways during spontaneous respiration, which can serve as a surrogate for native tissue mechanical properties [40]. These clinical modalities are critical to diagnose pulmonary disease and serve as important tools for disease surveillance and treatment response.

### 3.2. Micromechanical Properties of the Airway

The micromechanical properties of airway cells and extracellular compartment can define the mechanisms of repair, remodeling, and disease. This is typically quantified with the use of atomic force microscopy (AFM), a high-resolution imaging technique that permits not only nanometer-level resolution, but also the quantification of the stiffness (Young’s modulus) at the cellular level (Table 2) [41,42]. Atomic force microscopy has been used to measure the average fibril diameter of type I collagen (400 nm) and type II collagen (100 nm) [43]. In addition, AFM can be used to quantify micromechanical properties, generating matrix stiffness as an aggregate of collagens, elastin, and proteoglycans. While the Young’s modulus of type I collagen fibrils in tendons has been studied in numerous animal models (comparing type I collagen fibrils in tendons among species) [44,45,46,47,48], assessment of the micromechanical properties of types of collagen in the airway has not been profiled. As ECM homeostasis and collagen remodeling are dynamic, likely are the associated mechanical properties during these processes [49]. Therefore, these micromechanical properties can serve to elucidate the mechanisms of airway ECM remodeling and serve as a biomarker for disease development [50].

Within the airway, bronchial and alveolar cells have also been studied extensively using AFM. In vitro studies of wound repair demonstrate localized changes in cellular stiffness during bronchial epithelial spreading and migration [51]. Matrix micromechanics can also provide essential information to study cell–matrix mechanical crosstalk in organ biofabrication [52]. Micromechanical properties have elucidated the role of mechanotransduction in cell–matrix interactions, influencing proliferation, differentiation, and migration [53,54,55,56]. AFM has been used as a method of assessing the effects of decellularization. Decellularization preserved the elastic modulus in human lung slices; however, stiffness can be modulated by section thickness [52,55,57,58,59]. Current studies illuminate the heterogeneity of quantification using AFM; results can vary due to disease status, model, regions, and thickness of samples. Thus, a homogenous approach to test airway stiffness is necessary to compare analogous constructs. A comprehensive review of studies on airway mechanics and the quantification of airway stiffness is summarized in Table 2. The variability of these approaches suggests that a comprehensive approach including both macro- and micromechanical properties is needed to study airway mechanics.

**Table 2:** Young’s modulus of macro and micro quantification of airway cells, ECM, and tissue. AFM: atomic force microscopy.

Approaches	Tested Sample	Species	Types of Macromechanical Testing and Micro AFM Cantilever	Young’s Modulus (kPa) Mean ± SE (Unless Specified)	Ref
Macro Scale	trachea, bronchi	porcine	Displacement-controlled uniaxial tensile tests	The linear pseudo-elastic modulus (PEM, elastic response to an applied stress) of axial orientation (30.5 ± 3.1) was significantly higher than circumferential (8.4 ± 1.1); the circumferential PEM of small bronchi (12.5 ± 1.9) was higher than that of the trachea (6.0 ± 0.6) and large bronchi (6.6 ± 0.9).	[31]
	trachea	dolphin	Uniaxial compression test	65 ± 58 (SD)	[33]
	trachea	rabbit	Tensile test	13.6 ± 1.8 × 10^3^ for native 17.3 ± 3.5 × 10^3^ for decellularized trachea (SD)	[35]
	trachea	dog	Three-point bending test	proximal: 1.59 ± 0.24 × 10^3^, middle: 1.53 ± 0.42 × 10^3^, distal: 1.61 ± 0.22 × 10^3^	[32]
Micro Scale	Alveolar epithelial cells (A549)	Human	Silicon nitride triangular regular four-sided pyramid cantilevers, with a nominal semi-included angle θ = 35°, with nominal spring constant k = 0.01 N/m and 200-μm length (uncoated Microlevers, Thermomicroscopes, Sunnyvale, CA)	1.59 ± 0.33	[41]
	Bronchial epithelial cells (BEAS-2B)	Human		1.55 ± 0.41	[41]
	Type I lung epithelial	Rat	Standard blunt pyramidal tip silicon nitride cantilever with a nominal spring constant of 0.03 N/m	2.5 ± 1.0 (nucleus) 2.5 ± 1.2 (cytoplasm)	[42]
	Type II lung epithelial	Rat		3.1 ± 1.5 (nucleus) 4.7 ± 2.9 (cytoplasm)	[42]
	Lung fibroblast	Rat		3.3 ± 0.8 (nucleus) 6.0 ± 2.3 (cytoplasm)	[42]
	Bronchial epithelial cells (16HBE)	Human	Very soft cantilevers with spring constants of about 0.01 N/m and tip half-opening angle of ∼35° (Microlever/Sharp Microlever; TM Microscopes, Santa Clara, CA)	8.7 ± 0.23 Medians from wound: 2.4 kPa (0~10 µm), ~9 kPa (10~20 µm), 2.4 kPa (20~50 µm)	[51]
	Bronchus (400 μm)	Mice	Sphere-tipped probe (Novascan, Ames, IA) with a diameter of 5 μm and a nominal spring constant of ∼60 pN/nm probe	23.1 ± 14 (SD) (median 18.6 kPa)	[54]
	Lung tissue (1 mm)	Human	Spherical tipped-silicon nitride cantilever (Bruker, Camarillo, CA) with a 4.74-μm diameter and a 20–30 pN/nm spring constants probe	1.606 ± 0.08	[57]
	Decellularized lung matrices (1 mm)	Human		1.96 ± 0.13	[57]
	Lung parenchyma strips (400 μm)	Mice	Silicon nitride triangle cantilever with a 5-μm diameter borosilicate spherical tip, with a spring constant of 0.06 N/m probe (Novascan, Ames, IA).	Representative curves for bleomycin-treated lung: 13.39 kPa; for saline-treated lung: 0.732 kPa (median is localized, can be ~30 times higher comparing former t0 latter)	[55]
	Lung strips (100 μm)	Mice	Silica glass bead-customized silicon nitride AFM tip with diameter of 4.74 μm, and cantilever spring constants in the range of 0.06~0.08 N/m (Veeco, Plainview, NY)	Saline-treated mouse: 1.96 ± 1.21 (SD) Bleomycin-treated mouse (lung fibrosis model): 17.25 ± 11.06 (SD)	[58]
	Decellularized alveolar wall segments (∼7 μm)	Rat	A Si3N4 V-shaped Au-coated cantilever with a four-sided pyramidal tip on its apex with a semi-included effective angle (θ) of ∼20° and a nominal spring constant (k) of 0.1 N/m (MLCT, Bruker, Germany)	5.59 ± 3.39 (SD)	[59]
	Decellularized alveolar wall junctions (∼7 μm)	Rat		6.79 ± 3.88 (SD)	[59]
	Decellularized pleural membrane (∼7 μm)	Rat		15.76 ± 13.70 (SD)	[59]

## 4. The Role of Collagen in Airway Disease and Disease-Associated ECM Stiffness Change

In airway diseases, abnormal tissue remodeling is associated with the deposition of ECM components such as collagens, fibronectins, and proteoglycans, in and around the epithelium and surrounding vessels [60,61,62,63]. Pathologic collagen remodeling involves the reorientation and rearrangement of fibers in an effort to confer greater strength to the region of injury. With the high prevalence of collagen in the airway, its deposition or degradation is a surrogate for the stiffness change observed in airway disease. The pathogenesis and alteration of airway mechanics and collagen in pulmonary diseases and the aging lung are listed in Table 3. Burgeoning research in collagen homeostasis has the potential to identify biomarkers in the early diagnosis and treatment of lung diseases.

### 4.1. Increased Collagen Concentration in Cystic Fibrosis

Cystic fibrosis (CF) is an autosomal recessive disease that causes alterations in the cystic fibrosis transmembrane conductance regulator (CFTR) chloride ion channel, leading to thick mucus blocking the airway, causing infections and scarring of the lung [64]. This results in an alteration of cellular and matrix stiffness. Human epithelial cells derived from patients’ airways with CF and CFTR mutant cells have been found to have a lower Young’s modulus than normal human epithelial cells [65,66]. Alveolar matrix remodeling and fibrosis is present in the CF lung and leads to stiffening of alveolar tissues. In patients with CF, collagen I and elastin concentration in alveolar septa were increased ∼9-fold and ∼5-fold, respectively, as compared to healthy controls [67].

### 4.2. Collagen Deposition in Asthma

Asthma is chronic inflammation of the airway that leads to episodic narrowing of the airway, which is commonly exercise- or allergen-induced [68]. Over time, hyper-responsiveness of the airway leads to inflammation and airway remodeling. With airway remodeling in asthma, collagen deposition results in an increase in matrix stiffness [69,70]. Early in vivo studies on patients with asthma have found increased collagen at the bronchial submucosal level; increased deposition of type I, III, and V collagens in asthmatic airways is well established [69,71,72,73]. This pathologic collagen deposition contributes to fibrosis, which can contribute to disease progression and severity in asthma [74,75]. Beyond disease severity, genetic factors also play a role in matrix collagen content and subsequent lung mechanics [76].

The correlation between collagen deposition and ECM stiffness has also been studied in vitro. Human bronchial fibroblasts (HBF) derived from asthmatic patients had a higher elastic modulus compared to non-asthmatic HBF [77]. Asthmatic airway smooth muscle cells (ASMCs) secrete more collagen I and less collagen IV than non-asthmatic ASMCs [78]. ASMC-mediated collagen remodeling can be used to screen treatment to asthma by monitoring contraction and degradation of collagen [79]. In turn, when cultured in collagen substrates with higher stiffness (93 kPa) than control (23.1 kPa), ASMCs exhibited behaviors (e.g., stimulated proliferation) similar to asthma [54,80]. This suggests that the maintenance normal lung stiffness is essential to maintain native ASMC expression. Notably, myofibroblasts, an intermediate between fibroblasts and smooth muscle cells, and fibroblast-to-myofibroblast transition (FMT) contribute to progression of fibrosis in asthma. Transforming Growth Factor-Beta (TGF-β) induced FMT and ECM stiffness in asthma exits as a vicious cycle: increased ECM stiffness causes enhanced FMT, which in turn leads to increased secretion of collagen, resulting in a reciprocal increase in matrix stiffness [81,82,83,84]. Thus, the interruption of this cycle by decreasing collagen secretion or blocking FMT may be a target in future asthma therapeutics.

### 4.3. Enhanced Collagen Deposition in Idiopathic Pulmonary Fibrosis Is Associated with the Increased ECM Stiffness 

Idiopathic pulmonary fibrosis (IPF) is a progressive fibrosing interstitial pneumonia of unknown cause [85]. The incidence of IPF rises with age and carries a poor prognosis with a mean survival after diagnosis of 3 years [86]. Advances in defining the mechanisms of IPF describe a sequence of events that result in disease development: genetic predispositions, chronic epithelial cell turnover, and environmental exposures that ultimately lead to epithelial dysfunction [86]. Collagen has a prominent role in the pathogenesis of disease; deposits in the alveolar walls progressively destroy normal alveolar architecture [87,88]. From a mechanical perspective, decellularized and native IPF samples displayed higher stiffness than healthy lung samples [57,89]. The Young’s moduli derived from AFM and a low-load compression testing are listed in Table 3. Mass spectrometry revealed that the IPF acellular lung also exhibited a different matrisome profile (collection of ECM components/proteins) exclusively expressed type I, V, and XV collagens, and was composed of higher amounts of type III, IV, VIII and XIV collagens than normal tissue [57]. This suggests the role of increased collagen deposition in IPF is associated with the enhanced stiffness in the ECM. Homeostasis type I collagen is believed to have an essential role in IPF pathogenesis. TGF-β1 upregulates collagen I expression in fibroblasts cultured in 3D-collagen I gels [90]. Further, type I collagen upregulation was higher in fibroblasts derived from patients with IPF than from healthy controls [90]. The amount and stiffness of collagen fibers from IPF lung tissue were found to be similar to healthy tissue. However, lysyl oxidase (LOX) enzymes (responsible for collagen’s post-translational modification) were upregulated in primary human lung fibroblasts from patients with IPF. LOX inhibition normalized the dysregulated post-translational collagen cross-linking and reduced tissue stiffness [91]. Rather than increased deposition, Jones et al. believed that altered collagen architecture determined tissue stiffness in IPF [91].

### 4.4. Collagen I and III Are Remodeling Markers in COPD 

Chronic obstructive pulmonary disease (COPD) is an inflammatory disease of the lungs, manifesting as incomplete airflow obstruction resulting in emphysema and chronic bronchitis [92]. Typically resulting from tobacco smoke or other inhalational injury, COPD results from narrowing and inflammation of small airways as the emphysematous lung loses its elasticity, resulting in dyspnea, cough, and excessive sputum production. Innate and adaptive immune responses and disruptions in ECM remodeling result in airway and alveolar remodeling. In 2019, Ito et al. provided a comprehensive review of ECM change in COPD and the role of type I and III collagen as biomarkers for remodeling [8]. In patients with COPD, Kranenburg et al. demonstrated an increased expression of total collagens I, III, and IV in the basement membrane and an increased expression collagens I and III in bronchial lamina propria and adventitia [93].

As mentioned previously, emphysema is part of the pathophysiology of COPD [94]. ASMC proliferation is affected by ECM stiffness, resulting in smooth muscle loss and matrix softening in small and terminal airways of patients with emphysema [95]. Diseased lung presented higher collagen content and altered airway mechanics than normal lung, with lower dynamic tissue elastance as well as hysterisivity in a mouse model of emphysema [96]. Collagen fibers were found to be 24% thicker in rat lung with elastase-induced emphysema. In addition, the threshold of collagen to maintain mechanical stability is reduced, demonstrated by broken collagen fibers under similar stretch [97]. These findings suggest abnormal collagen remodeling has a significant role in COPD lung mechanics.

### 4.5. Collagen I and III Are Associated with Lung Mechanics Change in Acute Respiratory Distress Syndrome

Acute respiratory distress syndrome (ARDS) is a condition where the alveoli or alveolar vessels are injured, leading to inflammation and increased fluid in the alveoli. In patients with ARDS, mechanical ventilation can cause additional lung injury due to the barotrauma from high airway pressures [98,99]. In ARDS, collagens can serve as markers of remodeling in various regions of the airway [8]. Excessive type I and III collagens can be detected in interstitial edema. ARDS matrix remodeling in ARDS requires myofibroblast migration or contraction generating mechanical forces, which deposit type III collagen during the early stages of ARDS. In later stages of disease, there is an increase in type I collagen and collagenase-digested type III collagen, leading to a tendency towards fibrosis [8]. In animal models of early acute lung injury (a milder type of ARDS), tissue resistance and dynamic elastance increased in rat lung parenchymal strips. These mechanical properties were persistently high at the late stage; meanwhile, collagen fiber content increased exponentially with the injury’s severity [10].

### 4.6. Aging Is a Factor of Collagen Alteration in Lung

Lung function is known to deteriorate with age, resulting in poorer mucociliary clearance, loss of elastic recoil, and poorer lung function on PFT. One mechanism of lung aging is increased collagen and decreased elastin production by fibroblasts, thus increasing pulmonary stiffness and lowering compliance, increasing the elastic modulus [100,101]. In addition to changes of the quantity of certain matrix proteins, collagen undergoes post-translational modifications, increasing collagen cross-linking and thereby increasing rigidity while decreasing fiber length and width. These changes in collagen mechanical properties can influence response to therapeutics [102,103].

**Table 3:** Airway disease stiffness alteration and associated collagen change.

Airway Disease	Pathogenesis	Models	Related Stiffness Alteration	Collagen Change
Cystic fibrosis (CF)	Autosomal recessive mutation in a single gene that encodes the cystic fibrosis transmembrane conductance regulator (CFTR) protein, which leads to loss of chloride channel function [104,105].	Human bronchial epithelial cells (16HBE and CFBE) and lung tissue	Shorter cell topology and lower Young’s modulus of human epithelial cells from CF donors compared to controls [65,66] Median Young’s modulus of human CF bronchial epithelial cell is 61.19 MPa, ranging from 31.09 to 104.85 MPa, as compared to the 16HBE median of 79.81 MPa (range 36.51~144.64 MPa)	Type I collagen increased in alveolar septa of patients with CF versus tissues from healthy controls ~9-fold increase of collagen in patients with CF as compared to healthy controls [67]
Asthma	A chronic inflammatory disorder with hyper-responsiveness of the airway to different triggers [68]. TGF-β-induced fibroblast-to-myofibroblast transition [106].	Undifferentiated human bronchial fibroblasts (HBF) and airway smooth muscle cells (ASMCs) 2D and 3D culture models	Higher elastic modulus in asthmatic human bronchial fibroblasts (HBF) (18.75 ± 6.78 kPa) than normal HBF (5.39 ± 0.97 kPa) (mean ± SD) [77] ASMCs from asthmatics exhibited stiffer collagen substrate (93 kPa) than normal tissue (23.1 ± 14 (SD)) [54].	Deposited collagens I, III, and V increased in asthmatic airways [69]. Deposition of collagen types I and III in sub-epithelium was associated with asthma severity [74,75]. Excessive collagen deposits and increased airway stiffness were characteristic features of the pathological airway remodeling in asthma [107,108].
Idiopathic pulmonary fibrosis (IPF)	A progressive fibrosing interstitial pneumonia of unknown causes [85]	Lung tissue acellular model	Both native (16.52 ± 2.25 kPa) and decellularized (7.34 ± 0.6 kPa) IPF lung have higher Young’s modulus than normal lung (1.96 ± 0.13 kPa) [57]. Average IPF tissue stiffness (18.9 ± 11.1 kPa) was higher than healthy control (3.7 ± 1.3 kPa); Stiffness of hydrogel derived from IPF tissue (6.8 ± 2.8 kPa) was greater than hydrogel from healthy control (1.1 ± 0.2 kPa), as detected by a low-load compression tester [89].	Collagens I, V, and XV were expressed in IPF lung acellular matrix but not in healthy control; Collagens III, VI, VIII, and XIV were enriched in IPF matrices [57]. Excessive collagen deposition (mostly collage I) with active fibroblasts indicated active fibrosis in IPF [109]. Collagen architecture alteration was a deterrent to fibrosis [91].
Chronic obstructive pulmonary disease, (COPD)	An inflammatory disease of the lungs, manifesting as incomplete airflow obstruction resulting in emphysema and chronic bronchitis [92], mainly induced by smoke exposure [110].	ASMCs and lung tissue	COPD lung tissue (2.9 ± 0.8 kPa) and COPD ECM hydrogel (1.5 ± 0.4 kPa) had similar stiffness to healthy control by a low-load compression tester [89]. The dynamic tissue elastance was 19% lower and hysterisivity was 9% higher in emphysematous rat lung as compared to controls [96].	Increased expression of collagens I, III, and IV in the surface epithelial basement membrane Increased expression of collagens I and III in bronchial lamina propria) and adventitia [93] Collagen fibers in the emphysematous lung were 24% thicker than normal tissue [97]
Acute respiratory distress syndrome (ARDS)	Fluid accumulation in alveoli, with partial lung collapse (atelectasis) and low levels of oxygen in the blood (hypoxemia) [111].	Rat lung tissue	The ARDS lung is not stiff, as characterized by pressure–volume curves generated by computerized tomography (CT) at 3 levels of positive end-expiratory pressure (PEEP) [112]. High resistance and dynamic elastance in acute lung injury [10]	Myofibroblasts deposited collagen III in early stage Collagen I was synthesized while collagen III was digested by collagenase in the late stage [8]
Aging lung	Increased collagen and decreased elastin production by fibroblasts	3D matrix model and lung tissue	Increased pulmonary stiffness Decreased compliance	Collagen fibers have higher stiffness and lower deformation

## 5. Conclusions

As the major component of extracellular matrix (ECM), collagen supports the macro- and micromechanical environment of the airway. Preservation of collagen type and quantity is necessary to maintain ECM stiffness for the native airway as well as for the development of tissue-engineered airway constructs. Macro and micro airway mechanics provide a fundamental knowledge of the contribution of collagen in the airway. Biomechanical properties can serve as a reference for biomaterial creation and scaffold fabrication, can provide a diagnostic tool for airway disease, and can also elucidate the mechanisms of disease. Future directions for collagen mechanics include their application as a biomarker for disease surveillance and the development of therapeutics.

## Figures and Tables

**Table 1:** Role of the different types of collagen in airway.

Collagen Subtype	Collagen’s Role
Type I collagen	a primary contributor to lung mechanics provides mechanical stability and structure provides a structural framework in the bronchi, interstitium, and alveolar wall
Type II collagen	the major component of airway cartilage (95% of total collagen) facilitates chondrocyte synthesis of extracellular matrix (ECM)
Type III collagen	a primary contributor to lung mechanics provides a structural framework in the bronchi, interstitium, and alveolar wall
Type IV collagen	fundamental for maintaining the strength and function of the basement membranes
Collagen type I/type III ratio	determines the resistance of collagen fibers to breakdown under mechanical forces during stretching
